# Limitations of Environmental Extrapolation in Low-Cost Carbon Monoxide and Fine Particulate Matter Sensors

**DOI:** 10.3390/s26154988

**Published:** 2026-08-06

**Authors:** Sean Benjamin, Evan R. Coffey, Caroline Frischmon, Michael Hannigan

**Affiliations:** 1 Department of Mechanical Engineering, University of Colorado at Boulder, Boulder, CO 80301, USA; evan.coffey@colorado.edu (E.R.C.); caroline.frischmon@colorado.edu (C.F.); michael.hannigan@colorado.edu (M.H.); 2 Department of Environmental Engineering, University of Colorado at Boulder, Boulder, CO 80303, USA

**Keywords:** air quality, carbon monoxide, particulate matter, infiltration, low-cost sensors, calibration

## Abstract

**Highlights:**

Low-cost sensors must be calibrated to be accurate and are sensitive to variations in pollutant concentration, temperature, and humidity. Calibration model error increases as the model is extrapolated into pollutant concentration, temperature, and humidity environments past the range of data the model was trained on.

**What are the main findings?**

- Calibration model-biased error increases as it is extrapolated to data outside of the range the model was trained on.
- Calibration model random error increases as it is extrapolated to pollutant concentrations higher than what the model is trained on.

**What are the implications of the main findings?**

- Be cautious and transparent about measurement accuracy when extrapolating sensor calibration models outside of data ranges that those models were trained on.

**Abstract:**

Low-cost air quality sensors have grown in popularity. Calibrating these sensors in environments that perfectly mirror the environments they will be used in is difficult and can be costly, so researchers often need to extrapolate calibration models outside of the environmental and pollutant data ranges they are trained on. As such, understanding the impact that this extrapolation has on the resulting measurements is important when using this data. In this study we conducted a multi-season colocation between two low-cost sensors (Plantower PMS5003 for PM_2.5_ and Alphasense CO-B4 for CO) and Federal Equivalence Method monitors. We then trained multiple calibration models on different selective ranges of relative humidity, temperature, and pollutant concentration data to test how those models performed when extrapolated into data ranges. There were three main takeaways in this study. First, there was minimal bias error in calibration model performance when a model is applied to data that falls within its training data range. Second, extrapolating models into data that was outside of their training data ranges led to increases in bias error. Third, extrapolating models into higher pollutant concentrations than what the models were trained on increases random error.

## 1. Introduction

As people spend 90% of their time indoors, ensuring that the indoor environment is safe and does not adversely impact the health of its occupants is important [1,2]. Many studies investigate indoor air quality and its relation to the outdoor environment using low-cost sensors. Low-cost sensors are important for this kind of work as they enable dense air quality monitoring networks for a fraction of even one reference grade air quality monitor. Two air pollutants that are commonly investigated are carbon monoxide (CO) and particulate matter that is 2.5 µm or less in diameter (PM_2.5_), as they have been shown to cause adverse health effects [3,4,5,6]. When these pollutants are transported to residences, they can infiltrate homes and cause increased concentrations of pollutants in the indoor environment [7]. In the Hannigan Air Quality Research Lab, we monitor air pollutant concentrations inside and outside of houses to investigate the impact of emissions events using a suite of low-cost sensors (LCSs). As these measurements took place in Colorado from September to May, the outdoor and indoor environmental conditions, namely temperature and relative humidity, varied widely. While LCSs offer an attractive alternative to reference grade monitors, as they provide a way to investigate a range of pollutants in multiple locations for a fraction of the cost, LCS calibration is needed to provide accurate and precise air pollutant measurements [8,9,10,11]. Manufacturers often provide factory calibration models, but these have been shown to be inaccurate or inconvenient to use [12,13]. In situ calibrations are normally performed via colocations in which LCSs record sensor signals while next to reference-grade monitors, and then a regression is developed to fit the raw signal from the LCS to the measurements from the reference grade monitor [11]. Including relative humidity and temperature has proven to increase accuracy while reducing bias in multiple linear regression calibrations for these sensors [13,14,15]. Our team primarily conducts ambient colocations to sample calibration data from real-world testing environments at a low cost. However, this means that we do not control the temperature, relative humidity, or pollutant concentrations while sampling at the colocation site. Given that measurements collected in and around households (field measurements) between October and May have varied in temperature from −8 °C to 49 °C, relative humidity from 4% to 88%, and CO and PM_2.5_ concentrations of up to 5 ppm and 400 µg/m^3^, respectively, it would be infeasible to record every combination of pollutant concentration, temperature, and relative humidity in colocation that is seen in field measurements. The best option then is to extrapolate calibration models into data spaces outside of those which the models are trained on. In this study, we investigated the random error and systematic bias associated with extrapolating multiple linear regression (MLR) calibration models. By training calibration models on subsets of data from the colocation dataset, and then testing the performance of those models on the remaining data (data outside the training data subset), we compare the performance of these models to the reference monitor data to determine how accurately these models can be extrapolated into different data spaces. While there are many readily available LCS which use different physical mechanisms for measurements, the process for calibration extrapolation described here can be used to evaluate calibration models broadly.

## 2. Materials and Methods

For this study, we colocated air quality LCS packages with reference monitors at the Colorado Department of Public Health and the Environment (CDPHE) ambient air monitoring site at I-25 Denver throughout 2024. The colocation measurements nearly entirely encompass the temperature and humidity ranges seen in field data collection at houses near prescribed fires from 2023 to 2025. To investigate the importance of colocation overlap, we trained multiple calibration models on collections of the colocation data. These collections were grouped based on the lower, middle, or higher 20%, 40%, and 60% of each regression variable (temperature, relative humidity, or pollutant concentration). These models, developed on sorted and truncated datasets, were compared to baseline calibration models developed on randomly selected sets of data across the entirety of the calibration dataset, using the same quantity of data points as the respective 20%, 40%, or 60% groups. Additionally, we explored how these models performed under extrapolation. This process is explained in greater detail in Section 2.3.

### 2.1. Low-Cost Sensor (LCS) System

This study used custom LCS air quality monitoring packages called PODs. Two of the sensors used in these PODs are the Plantower PMS5003 (Plantower) particulate matter sensor (Nanchang Pateng Technology Co., Ltd., Nanchang, China). and the Alphasense CO-B4 carbon monoxide sensors (Alphasense Ltd., Great Notley, United Kingdom). The Plantower utilizes 90-degree light scattering to measure incident particle concentrations. The Plantower estimates particle sizes and separates them into 5 size bins to calculate PM concentrations. These size bins include particles greater than 0.3 µm, 0.5 µm, 1.0 µm, 2.5 µm, 5.0 µm, and 10 µm in diameter. According to the Plantower specification sheet, the particle counting efficiency is 50% for the size bin of 0.3 µm and 98% for the remaining size bins. The Plantower uses these particle counts to calculate mass concentrations of PM_1.0_, PM_2.5_, and PM_10.0_, using a black box model [16,17]. Some studies investigate this modeling and suggest that the Plantower responds to groups of particles similarly to a nephelometer [18,19]. A further study of the photodiode response in the Plantower states that these PM sensors are imperfect particle counters that respond to single light scattering events [20]. The Plantower does not have an optical focusing device between the scattered light and the photodiode to get a perfect cosine wave response. However, the photodiode responds to individual light scattering pulses and widths and assigns the particle count to a size bin based on this amplitude, which is more like an optical particle counter than a nephelometer. The Plantower sensor shows a response to varying relative humidity levels depending on the type of aerosol it is measuring [20,21,22,23]. This is due to the impact of humidity on hygroscopic aerosols, which can cause particles to grow in size as ambient water vapor attaches to particles. Hygroscopic growth causes the Plantower to sort the particles into larger-diameter bins, causing the internal concentration predictor to increase the PM concentration readings while the actual pollutant mass remains constant. There is also a reported response to temperature variations [22,23]. Temperature affects the air density which could impact the air sample flow through the device as well as optical properties.

The Alphasense CO-B4 sensor is an electrochemical sensor in which CO diffuses through the sensor’s membrane at a rate proportional to the gas concentration. The gas then reaches the anode and undergoes an oxidation reaction which will produce protons that travel through the sensor’s electrolyte, and electrons that flow through an external circuit. This external flow is measured and is linearly proportional to the gas concentration. This process is influenced by both temperature and humidity. According to Fick’s law, the rate of diffusion of a gas will increase with temperature, and so higher temperatures may be falsely associated with higher concentrations. Low humidity may also adversely impact the signal from the electrochemical sensor by causing a dryer electrolyte, inhibiting proton transfer. Additionally, high humidity can flood the electrolyte and cause signal instability. The datasheet provided by the manufacturer shows that the CO-B4 sensor is less responsive to pollutant concentration changes (40–70% of normal sensitivity) at low temperatures (−20 °C) and more responsive (110–125% sensitivity) at higher temperatures (50 °C) [24]. The Alphasense factory algorithm utilizes the auxiliary signal in the CO-B4 to attempt to correct for this temperature sensitivity. However, research has shown that this model can be improved with multiple linear regression (MLR) that includes both temperature and humidity [16,25]. There is also research indicating that an MLR model is sufficient to translate the sensor’s response to pollutants [26]. MLR was chosen for this study due to a blend of ease of implementation and low computational intensity, though other machine learning algorithms, such as gradient boosting, can be more accurate [27]. Gradient boosting is a nonlinear machine learning method shown to improve calibration fits for low-cost sensors [28,29]. As an ensemble tree-based method, gradient boosting is similar to the more commonly used random forest method, though it uses a “boosting” technique to build decision trees sequentially, improving upon the error of each prior tree [30]. To provide a small sample of nonlinear machine learning performance, gradient boosting (GB) models were developed for some data combinations, though the focus of this study is specifically on multiple linear regression for previously stated reasons. We chose gradient boosting as a nonlinear model comparison because of its improved ability to extrapolate extreme concentrations compared to random forest [29]. We built the gradient boosting models using the Scikit-learn module in Python (Version 3.12.4). Hyperparameters (learning_rate, n_estimators, max_depth, min_samples_split, min_samples_leaf, max_features, subsample in Scikit-learn) were tuned using the k-fold cross validation process described in Section 2.3.

### 2.2. LCS System Colocation

**Figure 1:**
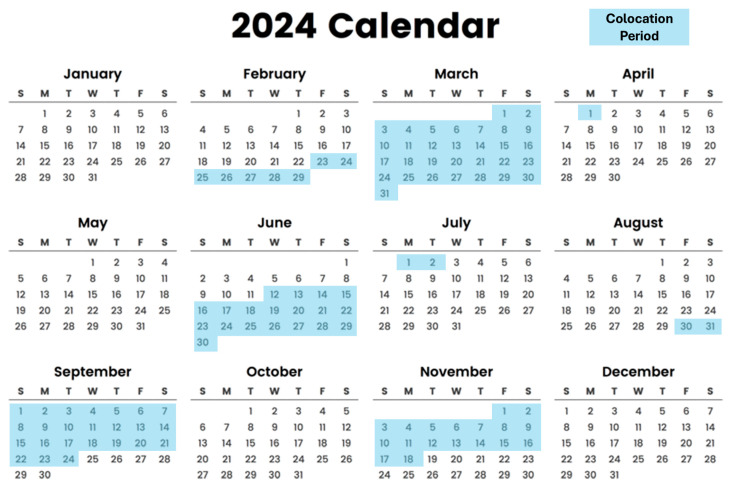
This is a calendar depicting the periods of colocating POD A with reference monitors.

The PODs were colocated at the CDPHE I-25 Denver site (AQS 08-031-0027) for a total of 104 days over the course of the 2024 calendar year (Figure 1). The PM reference monitor used in this study was the GRIMM EDM-180 (GRIMM). This reference monitor uses light scattering to measure particle size and concentrations similar to the Plantower [31]. The GRIMM EDM-180 (GRIMM Aerosol Technik, Ainring, Germany) is a certified federal equivalence method (FEM) continuous measurement monitor. FEM monitoring is a standardized method of air pollutant concentration monitoring, equivalent to Federal Reference Method (FRM) monitoring [32]. The CO reference monitor used in this study is the Thermo Fisher Scientific Model 48i-TLE Enhanced Trace Level CO Analyzer (TSM) (Thermo Fisher Scientific Inc., Waltham, MA, USA), which is a FEM monitor. The TSM uses gas filter correlation, non-dispersive infrared, and sample temperature stabilization to measure CO with a low-concentration limit of detection [33]. The monitor measures infrared absorption of both CO and nitrogen gas (N_2_) for side-by-side comparison. This is a certified FEM continuous measurement monitor [32]. Both reference monitors monitor climate control samples (temperature and humidity) at the intake to prevent interference.

### 2.3. LCS System Calibration

As colocations are time- and resource-intensive, we investigated whether narrower ranges of environmental conditions or pollutant concentrations in calibration datasets affected model performance; in other words, what change in model performance do we see when we extrapolate from a model’s calibration data space and will this improve with additional colocation?

**Figure 2:**
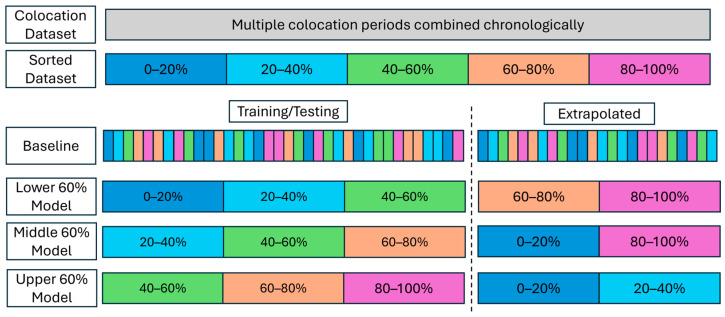
The colocation dataset was copied and sorted for CO, PM_2.5_, temperature, and humidity. These sorted datasets were then truncated into lower, middle, and upper 20%, 40%, and 60% groups later used for calibration model generation. This figure shows an example of 60% truncation for each of the lower, middle, and upper model of any given sorted dataset.

To prepare data for analysis, first, we time averaged each set of data to 5 min median values. After time averaging, there were 28,798 data points from POD A and 19,246 data points from POD B, as shown in Table 1. Next, we created 4 copies of our colocation dataset: one sorted by temperature, one sorted by relative humidity, one sorted by the reference monitor’s PM concentration, and one sorted by the reference monitor’s CO concentration. Each of these four sets were truncated into 20%, 40%, or 60% data groups corresponding to the lower, middle, and higher percentiles. The 20%, 40%, and 60% truncation groups used 5759, 11,519, and 17,279 data points respectively in POD A and 3849, 7698, and 11,547 data points respectively in POD B, as shown in Table 1. An example of the sorting and truncation process is shown in Figure 2.

**Figure 3:**
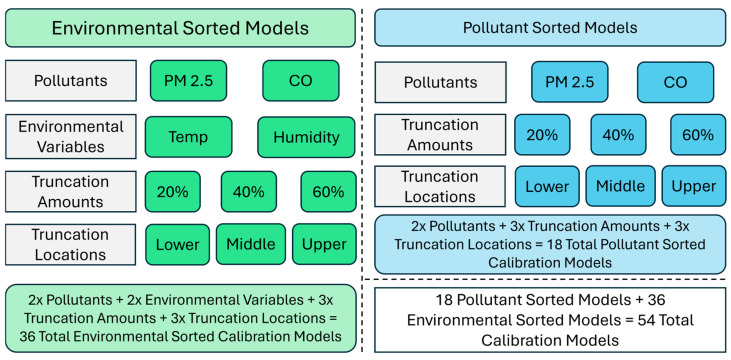
There are 54 total sorted calibration models from sorted pollutant concentration and environmental condition datasets.

Sorting and truncating the colocation dataset created 36 groups of data, 18 of which were environmentally sorted (i.e., 2 variables [temperature and RH] × 3 levels [lower, middle, upper] × 3 percentiles [20%, 40%, 60%] = 18). Those 18 environmentally sorted groups were used to create one PM_2.5_ calibration model each, and one CO calibration model each. An additional 18 groups came from pollutant-sorted data based on CO and PM_2.5_ concentrations (2 pollutants × 3 levels [lower, middle, upper] × 3 percentiles [20%, 40%, 60%] = 18). There were then 54 total groups of data to develop calibration models as seen in Figure 3.

**Figure 4:**
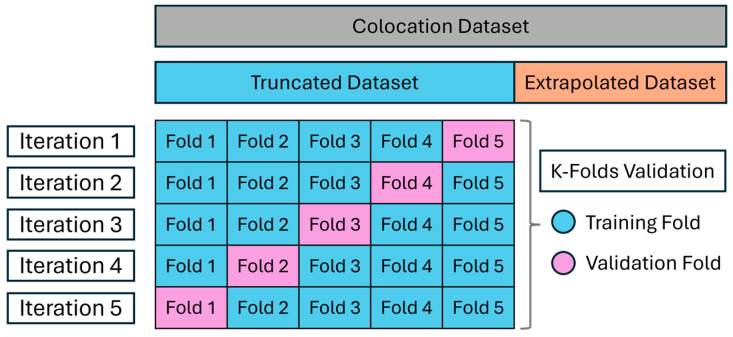
K-Fold Cross Validation (where K = 5)—MLR calibration model generation and choice methodology. Data points were divided randomly into 5 equally sized groups which were iteratively combined 5 times into sets of 4 training groups and 1 validation group. A model was generated on each combination of training groups. We chose the calibration model based on best performance with the validated group.

Second, we z-scored the sensor signal, the temperature, and the relative humidity. This accounted for signal variability between individual sensors as shown by Okorn et al. [11]. Lastly, 5-fold cross validation was performed by randomly partitioning each of the 54 datasets into 5 equally sized subsets of data (Figure 4). Five MLR models were trained, each using four subsets for training and the remaining subset for validation. The model with the lowest validation root mean squared error (RMSE) was selected as the calibration model for that dataset.

Various mathematical techniques can be used to translate LCS signals to pollutant concentrations by relating those signals to reference monitor measurements. Many different machine learning methods such as artificial neural networks and random forest modeling have been used to develop calibration models with varying success; however, we employed multiple linear regression due to its simplicity, computational efficiency, and high accuracy [26,27,34].

The MLR equation is in the form of Equation (1) for the Plantower PMS5003

(1) $$PM_{2.5}=a_{1}*P{M_{2.5}}_{PT}+b_{1}*T+c_{1}*RH+d_{1},$$

and Equation (2) for the Alphasense CO-B4

(2) $$CO=a_{2}*CO_{Main}+b_{2}*CO_{Aux}+c_{2}*T+d_{2}*RH+e$$

where *a*, *b*, *c*, *d*, and *e* are the regression constants assigned by the respective MLR. PM_2.5_ is the calibrated PM_2.5_ concentration, and CO is the CO concentration from the reference instrument. $P{M_{2.5}}_{PT}$ is the PM_2.5_ concentration estimate from the Plantower (data field 2, PM_2.5_ CF = 1 particle concentration), *T* is the temperature from the POD, and *RH* is the relative humidity from the POD. $CO_{Main}$ is the main concentration signal from the Alphasense CO-B4, while $CO_{Aux}$ is the auxiliary signal used for temperature correction in the Alphasense CO-B4 factory algorithm. The PM_2.5_ signal from the PMS5003 uses the sensor’s internally calculated PM_2.5_ concentration with no additional correction factor (CF = 1). We used the MATLAB R2021b “fitlm” function which uses least squares to determine coefficients for an equation to relate the combination of LCS data to reference data (MATLAB is from MathWorks, Natick, MA, USA).

A baseline model for each POD was also created for comparison, where the three-step process was performed on the complete colocation dataset instead of just the truncated data. These models are referred to as “Overall Baseline Models.” Additionally, a baseline model was created using the above three-step process above for a dataset equivalent to 20%, 40%, and 60% of the total colocation dataset, where each group comprised randomly selected data points across the entire colocation dataset. This resulted in a total of 6 Truncated Baseline Models for each POD (20%, 40%, 60% for CO; 20%, 40%, 60% for PM_2.5_).

For each truncated dataset, the data from the colocation dataset outside of the truncation were also saved. This data is referred to as the respective truncation’s extrapolated dataset. Each model was applied to its respective extrapolated dataset to understand model performance in conditions not included in training. For example, the lower-20% temperature dataset was truncated to contain data measurements associated with the coldest 20% of temperature measurements. Data not included in this truncated dataset, or the warmest 80% of measurements, comprised the extrapolated data. The model developed on the truncated data was extrapolated (applied to this extrapolated dataset) to see how the model performed on data that it was not trained on. Each model’s performance was tested on its respective extrapolated dataset for evaluation.

We used Mean Bias Error (MBE) to investigate the bias error associated with the calibration models:

(3) $$MBE=\frac{1}{n}\sum_{i=1}^{n}\hat{y_{i}}-y_{i}$$

It is important to minimize bias so that we can compare measurements from multiple LCS packages across field deployments. Root mean squared error (RMSE) is the average magnitude of error associated with each model:

(4) $$RMSE=\sqrt{\frac{1}{n}\sum_{i=1}^{n}{\left(\hat{y_{i}}-y_{i}\right)}^{2}}$$

However, RMSE is affected by bias so we also used Centered Root Mean Square Error (CRMSE) to investigate the precision associated with the calibration models:

(5) $$CRMSE=\sqrt{RMSE^{2}-MBE^{2}}$$

Evaluating the models by using CRMSE allows for the investigation of pattern similarity between models and reference data, specifically correlation and amplitude rather than constant offsets occurring from bias. In Equations (3)–(5), *n* is the number of observations being investigated, $\hat{y}$ is the predicted value given by the investigated calibration function, and *y* is reference monitor value.

### 2.4. Performance Reproducibility

**Figure 5:**
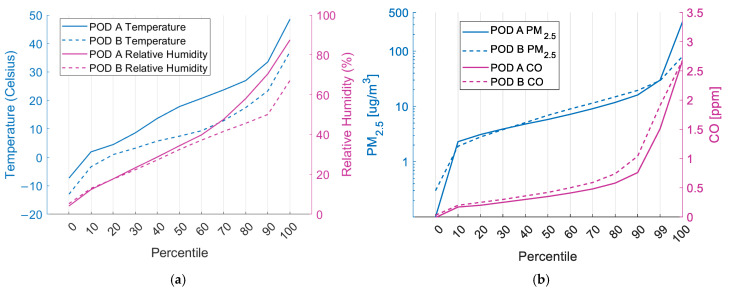
Colocation measurements for PODs A and B. These plots show the difference in concentration or environmental measurement. (**a**) Comparison of environmental condition percentiles. Temperature and relative humidity measurements come from the PODs’ BME 680 sensor. (**b**) Comparison of pollutant concentration percentiles during colocation. Note that the PM_2.5_ concentration axis is in log-scale.

We investigated the reproducibility of this study by completing the same model development across two PODs, POD A and POD B. PODs A and B shared the same colocation site, but did not share the full time range of collocation. As a result, the edges of the percentile data are similar between PODs but not exact, as seen in Figure 5. Specifically, the PM_2.5_ concentrations seen by POD A and B at the 100th percentile differ greatly despite the PODs sharing the same 99th percentile concentration. This large spike could affect both models trained on this data, and the performance of models trained on other data but tested against this higher concentration. We investigate this effect later in the results and discussion. The number of total measurements is also slightly different between PODs, as seen in Table 1.

## 3. Results

In this section, we review the performance of the models in their respective truncated and extrapolated data spaces using MBE vs. CRMSE plots similar to Okorn et al. [11]. Previous studies have shown that MLR models for Plantower PMS5003 have an RMSE of 3.4–7 [µg/m^3^] and the CO-B4 has an RMSE of around 61 ppb [25,27,35,36,37,38]. While MBE was less consistently investigated, an MBE of −5.81% ± 32.11% to 10.18% ± 44.88% was reported for PM_2.5_ and a mean absolute error of less than 40.6 ppb and above −32.4 ppb was report for CO. The baseline calibration models for the PODs in this study had an RMSE of 5.53 and 2.85 µg/m^3^ for PM_2.5_ and 200 and 130 ppb for CO. These baseline models also produced an MBE of −0.55 and −0.05 µg/m^3^ for PM_2.5_ and −11.5 and 0.29 ppb for CO. While CRMSE was not previously investigated in many studies, investigating a combination of CRMSE and MBE helps us understand the nuance of different types of error in calibration models.

**Figure 6:**
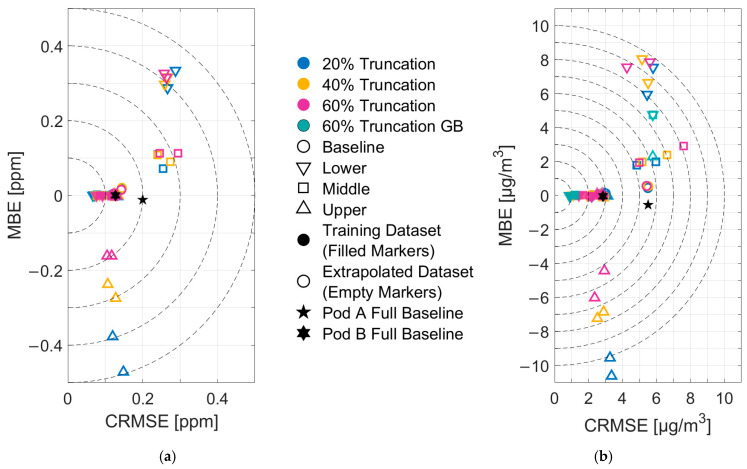
Performance of calibration models trained on different subsections of data selected from a pollutant concentration sorted dataset. Each point represents the MBE and CRMSE of a different calibration model in designated data spaces. Solid points represent the calibration model performance on a data space that is within the bounds of the truncation that was used for training the model, but not the data that was used in its training. The empty points represent the calibration model performance on data that falls outside of the bounds of that model’s truncation space. There is a point for each combination of truncation percentage, data space (truncated or extrapolated), and truncated data location within the sorted dataset (lower, middle, upper, and POD). Baseline models, which are trained on randomly selected data from across the entire non-truncated dataset, are included from each POD for comparison. Tabulated model performances can be found in the Supplemental Materials in Table S1 (CO-sorted models) and Table S2 (PM_2.5_-sorted models). (**a**) Performance comparison of the CO concentration-sorted calibration models. (**b**) Performance comparison of the PM_2.5_ concentration sorted calibration models. This subfigure includes 3 gradient boosting (GB) models represented in teal.

**Figure 7:**
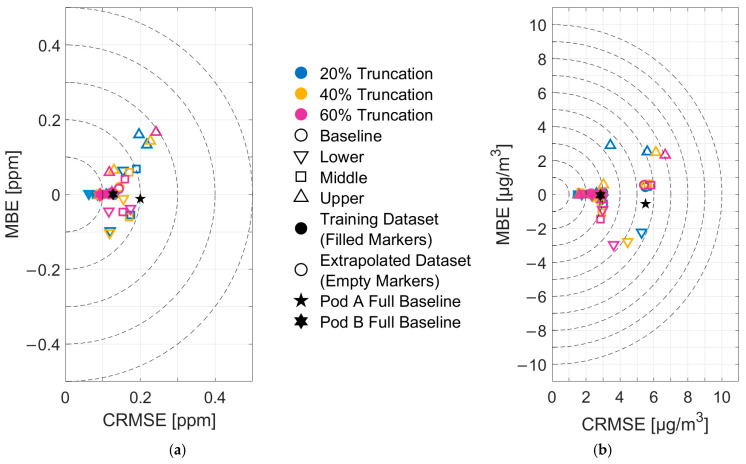
Performance of calibration models trained on different subsections of data selected from a temperature-sorted dataset. Each point represents the performance of a different calibration model in designated data spaces. Solid points represent the calibration model performance on a data space that is within the bounds of the truncation that was used for training the model, but not the data that was used in its training. The empty points represent the calibration model performance on data that falls outside of the bounds of that model’s truncation space. There is a point for each combination of truncation percentage, data space (truncated or extrapolated), and truncated data location within the sorted dataset (lower, middle, upper, and POD). Baseline models, which are trained on randomly selected data from across the entire non-truncated dataset, are included from each POD for comparison. Tabulated model performances can be found in the Supplemental Materials as Table S3 (temperature-sorted CO models) and Table S4 (temperature-sorted PM_2.5_ models). (**a**) This subfigure is the performance of the CO calibration models. (**b**) This subfigure is the performance of the PM_2.5_ calibration models.

**Figure 8:**
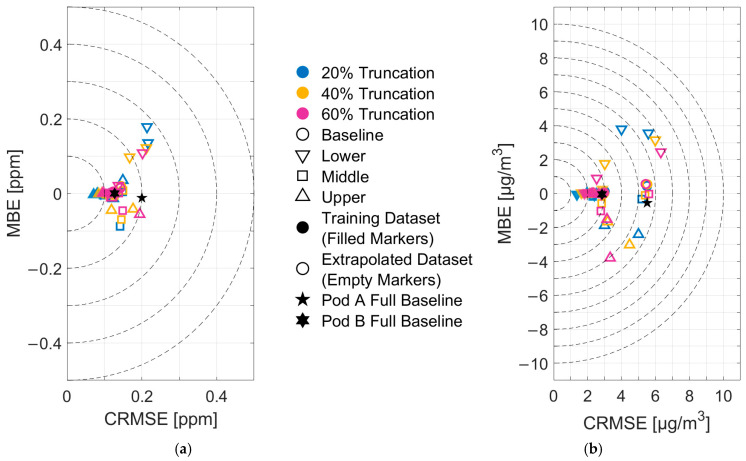
Performance of calibration models trained on different subsections of data selected from a relative humidity-sorted dataset. Each point represents the performance of a different calibration model in designated data spaces. Solid points represent the calibration model performance on a data space that is within the bounds of the truncation that was used for training the model, but not the data that was used in its training. The empty points represent the calibration model performance on data that falls outside of the bounds of that model’s truncation space. There is a point for each combination of truncation percentage, data space (truncated or extrapolated), and truncated data location within the sorted dataset (lower, middle, upper, and POD). Baseline models, which are trained on randomly selected data from across the entire non-truncated dataset, are included from each POD for comparison. Tabulated model performances can be found in the Supplemental Materials as Table S5 (RH-sorted CO models) and Table S6 (RH-sorted PM_2.5_ models). (**a**) This subfigure is the performance of the CO calibration models. (**b**) This subfigure is the performance of the PM_2.5_ calibration models.

In Figure 6, Figure 7 and Figure 8 various model performances are displayed. The color of each point represents the amount of truncation performed on the sorted dataset to create a training dataset, and the shape of the point represents where from the sorted dataset the truncation occurred. The filled data points represent the model performance in the truncated dataset, and the empty points represent respective model performance in the extrapolated dataset. For example, in Figure 6a, the empty yellow squares represent how, for each POD, the model trained on the middle 40% of PM_2.5_ concentrations performed when applied to the remaining 60% of the colocation dataset that was not used in training. Each figure also has 1:1 MBE:CRMSE zones marked by black dashed lines. These lines help in comparing model performances as points on the same dashed line sharing the same RMSE.

### 3.1. Pollutant Concentration-Based Calibration and Extrapolation

The first set of models that we investigated were those trained on pollutant concentration-sorted datasets. The models that were trained on datasets sorted by PM_2.5_ concentration are seen in Figure 6b. As a short guide for how to look through this figure, we will walk through the lower 60% PM_2.5_ concentration model. For this group, we applied the model to the data within the range of data used for training the model, but not used on training the actual model; this is 20% of the data within the lower 60% of pollutant concentrations collected during colocation, which is randomly selected across that lower 60% range. The model’s performance on fitting this data is called the truncated lower 60% PM_2.5_ model and is represented by a filled (truncated) pink (60%) upside-down triangle (lower). This model is also extrapolated outside of the range of its training data, which was into the highest 40% of PM_2.5_ concentrations. The performance of this model is called the extrapolated lower 60% PM_2.5_ model and is represented by an empty (extrapolated) pink (60%) upside-down triangle (lower). The truncated lower 60% PM_2.5_ model has a CRMSE of 1.43 µg/m^3^ and an MBE of 0.01 µg/m^3^ for POD A, and a CRMSE of 2.17 µg/m^3^ and an MBE of −0.01 µg/m^3^ for POD B, indicating both relatively low random error and bias. The extrapolated lower 60% PM_2.5_ model has a CRMSE of 5.61 µg/m^3^ and an MBE of 7.86 µg/m^3^ for POD A, and a CRMSE of 4.25 µg/m^3^ and an MBE of 7.56 µg/m^3^ for POD B, indicating that random error and bias grow for this model as it is extrapolated outside of the range of data used in training the model, and the model will overestimate compared to an FEM monitor. The truncated models outperformed the overall baseline models in both random and bias error, while the extrapolated models had competitive random error but much more bias error.

The same process works for investigating CO models in Figure 6a. The model developed on the lower 60% of CO concentrations from the colocation period is called the lower 60% CO model. When this calibration model is applied to data within the range of its training data bounds, but not that exact data, it is referred to as the truncated lower 60% CO model. When the calibration model is applied to the data outside of the range of its training data, or the highest 40% of CO concentrations from the colocation period, it is called the extrapolated lower 60% CO model. Both models have pink upside-down triangles on the plot but the truncated model point is filled and the extrapolated model point is empty. The truncated model CRMSE is 76 ppb and the MBE is 0. 07 ppb for POD A, and 92 ppb and −0.55 ppb, respectively, for POD B, while the extrapolated model CRMSE is 263 ppb and the MBE is 315 ppb for POD A and 256 ppb and 327 ppb, respectively, for POD B. Similar to the PM_2.5_ performances, the extrapolated models have higher errors compared to the models that are only applied within the data bounds of their training data, and they tend to overestimate pollutant concentrations compared to the FEM monitor. These models also shared the same relation to the overall baseline models as the PM_2.5_ concentration-sorted models.

Generally, the most apparent takeaway from the concentration-sorted models shown in Figure 6 was the separation between the model performances in the truncated and extrapolated datasets. The truncated models all performed similarly with CRMSE values between 1 and 3 μg/m^3^ or 0.05 and 0.15 ppm. The CRMSE increased when the models were applied to the extrapolated data space instead of the truncated data space, except for the models trained on the highest concentrations (upper), indicating that missing peak concentrations in training data increased this random error. Models generated using middle truncated datasets had the lowest absolute MBE when the models were applied to the extrapolated datasets. The lower truncated models overestimated, and the upper truncated models underestimated relative to the reference. Extrapolation into higher or lower concentrations resulted in small relative increases in random error but relatively large increases in bias.

In addition, Figure 6b includes gradient boosting models developed on the lower, middle, and upper 60% of PM_2.5_ concentrations. While the middle 60% and lower 60% extrapolated models (those extrapolating up) out-perform their respective extrapolated MLR counterparts, the upper 60% extrapolated model has a similar RMSE to the extrapolated MLR counterpart, though with a larger CRMSE and a lower absolute bias error. Each truncated gradient boosting model outperforms its respective truncated MLR counterpart.

One of the main differences between CO- and PM_2.5_-based models is the grouping of the overall baseline models. The CRMSE for the PM_2.5_ overall baseline model for POD A is much higher than that of POD B. The CO baseline models share a tighter grouping than the PM_2.5_ models, having near identical CRMSE values. This indicates that the PM_2.5_ models are trained on differing pollutant concentrations where one contains a larger portion of high or low concentrations. The highest PM_2.5_ concentrations are higher in POD A than in POD B as seen in Figure 5b.

### 3.2. Temperature and Relative Humidity-Based Calibration and Extrapolation

The performance of the CO and PM_2.5_ calibration models trained on truncated temperature groups is shown in Figure 7 and the performance of the CO and PM_2.5_ calibration models trained on truncated relative humidity groups is shown in Figure 8. General trends from pollutant concentration-based models continue in environmental conditions-based calibration models where random error and bias for both the CO and PM_2.5_ calibration models increase when models are extrapolated into data ranges outside of what they were trained on.

Performance changes between models in truncated and extrapolated environmental data ranges have much lower relative changes than in the pollutant concentration data ranges. The maximum bias added for CO models when extrapolating was 0.18 ppm from the model trained on the lowest 20% relative humidity and 0.16 ppm from the model trained on the lowest 20% temperature, while the maximum random error added was 0.13 ppm in both the lower 20% relative humidity and upper 20% temperature. These are much lower than the pollutant concentration-sorted models where the maximum added bias for CO was 0.47 ppm and the added random error was 0.21 ppm, which are at least 0.29 ppm and 0.08 ppm greater than the environmental condition-based error measurements, respectively. The maximum bias added for PM_2.5_ models when extrapolating was 3.87 µg/m^3^ from the model trained on the lowest 20% relative humidity and 2.37 µg/m^3^ for the model trained on the upper 20% temperature, while the maximum random error added was 4.50 µg/m^3^ from the model trained on the lowest 60% relative humidity and 5.06 µg/m^3^ from the model trained on the upper 60% temperature. These are much lower than the pollutant concentration-sorted models where the maximum added bias for PM_2.5_ was 10.7 µg/m^3^ and the added random error was 5.93 µg/m^3^, which are at least 6.83 µg/m^3^ and 0.87 µg/m^3^ greater than the environmental condition-based error measurements, respectively.

In general, all extrapolation, in temperature and humidity, adds random error and bias for both the CO and PM_2.5_ calibration models. In other words, the truncated models have lower absolute MBE and CRMSE compared to the performance of the same models in extrapolated data ranges. In temperature-sorted models, models trained on higher temperatures tend to overestimate pollutant concentrations. Models trained on lower or middle temperatures tend to underestimate pollutant concentrations, though this is not a consistent trend as some of these models will also overestimate. In the relative humidity-sorted models, the lower models, or those trained on data from the driest conditions, tend to overestimate pollutant concentrations. The upper models, or those trained in the most humid conditions, tend to underestimate pollutant concentrations, though some of these models have an MBE closer to zero or even positive. The models trained on middle relative humidity values perform better than the upper or lower models in terms of bias.

## 4. Discussion

Both pollutant concentrations and environmental conditions affect low-cost sensor signal, and thus the performance of calibration models. Analyzing the performance of 54 different calibration models across varying sets of data both within and outside of their respective training data ranges provides a few high level takeaways. First, there is minimal bias error in calibration model performance when that model is applied to data that falls within the range of data included in the training data range. This can be seen in Figure 6, Figure 7 and Figure 8 when comparing the truncated and extrapolated models. Second, extrapolating models into data ranges outside of what they were trained on primarily introduces bias error. This is seen in the difference between filled and open points on Figure 6, Figure 7 and Figure 8 and the added bias is larger in sorted pollutant concentration-based models than sorted temperature or relative humidity-based models. Third, extrapolating models into higher pollutant concentrations than what the models were trained on increases random error. This is seen in Figure 6, with models trained on the middle or lower pollutant concentrations. When extrapolating models trained on higher pollutant concentrations to lower pollutant concentration data ranges, the models tend to underestimate concentrations but have relatively low random error, similar to when models are applied to data that falls within their training data range. It is important to note that temperature, humidity, and pollutant concentrations are not independent, and high pollutant concentrations may occur exclusively within higher or lower temperature or humidity ranges in a given dataset. As such, when extrapolating, one must be mindful of environmental parameter space, as extrapolating into one unfamiliar data space may also mean extrapolating into another (i.e., extrapolating into high pollutant concentration and high temperature at the same time). Supplemental Figure S2 shows an example of how this trend may occur in the POD 1 dataset.

Let us drill into those first two takeaways which focus on bias. The average absolute bias in CO model performance within its training data range is 0.001 ppm, while the average absolute bias for PM_2.5_ is 0.041 µg/m^3^. Additionally, overall baseline models, which also test performance in data ranges overlapped by the training data range, have an average absolute bias error of 0.006 ppm and 0.2985 µg/m^3^ for CO and PM_2.5_ respectively. When the temperature-sorted models are extrapolated, their average absolute bias error is 0.079 ppm and 1.41 µg/m^3^ for CO and PM_2.5_ respectively, and when the relative humidity-sorted models are extrapolated their average absolute bias error is 0.060 ppm and 1.78 µg/m^3^ respectively. These environmentally sorted models have an absolute bias increase when extrapolated by nearly 15 and 20 times their respective truncated models. This trend continues when the pollutant concentration-sorted models are extrapolated into data ranges outside of what they were trained on, as the average absolute bias is 0.232 ppm for CO and 5.63 µg/m^3^ for PM_2.5_, which is nearly 4 and 5 times larger than the environmental counterparts or nearly 60 times larger than the respective truncated models.

Now, let us drill into the third takeaway. Extrapolating models into pollutant concentrations outside of what the model was trained on adds both bias and random error; however, random error is only significantly increased when extrapolating into higher concentrations rather than into lower concentrations. The average random error for models tested on data that falls within their respective training data ranges were 0.101 ppm for CO and 2.12 µg/m^3^ for PM_2.5_. The average random error when extrapolating the models trained on higher pollutant concentrations into data that falls below the training data range was 0.121 ppm for CO and 2.88 µg/m^3^ for PM_2.5_. The average random error when extrapolating both upward and downward into pollutant concentrations outside of the training data range was 0.267 ppm for CO and 5.29 µg/m^3^ for PM_2.5_. The average random error when extrapolating just into pollutant concentrations larger than what the respective model was trained on was 0.258 ppm for CO and 5.86 µg/m^3^ for PM_2.5_. As such, random error for the models extrapolating into pollutant concentrations higher than the data range they were trained on were nearly double the random error for models that extrapolated into lower concentrations or that were tested in pollutant concentrations that fell within their respective training data ranges. This indicates that we have a difficult time fitting pollutant concentration peaks. This trend has been discussed by Frischmon and Silberstein along with possible solutions [39]. One solution is through utilizing other machine learning algorithms. Gradient boosting could be more effective than MLR for minimizing the bias associated with extrapolation. Comparative examples of gradient boosting model performance are shown alongside MLR performance in Figure 6b.

Other researchers can incorporate these results into LCS work to help them prepare for calibration work and/or provide them with further context for data analysis. Extrapolating calibration models into data spaces that are not well represented in their respective training datasets can result in higher random error and bias; and this study demonstrates the direction and magnitude of those increases. For example, in our work we understand the range of pollutant concentrations from the colocation data does not perfectly represent the range of pollutant concentrations that are anticipated near households due to prescribed fire emissions. This will result in extrapolation beyond the calibration models into a concentration data space that is not well represented in the data used to train the calibration model. As such, we anticipate that, on average, our predicted PM_2.5_ concentrations will be between 3 and 40 µg/m^3^, and CO will be between 0.3 and 2.5 ppm. While these pollutant concentrations largely fall within the full range of pollutant concentrations seen during colocation (see Figure 5b), we also expect to see pollutant concentrations above these ranges due to pollutant enhancement events such as smoke from cooking or a prescribed fire. In such cases, our calibration model would be behaving similarly to a lower extrapolation model, specifically the extrapolated lower 60% PM_2.5_ or CO models. Given that calibration models tend to overestimate when extrapolated into pollutant concentrations above the upper bound of their training data range, we will likely be overestimating our peak concentrations as well as observing an increased random error. If we leverage our 60% concentration extrapolation observations in Figure 6, in those extrapolation ranges we will be overestimating PM_2.5_ concentrations by 6–9 µg/m^3^ and CO concentrations by 0.3–0.4 ppm, as well as roughly doubling our random error. As researchers, we will need to keep both of these errors in mind as we make comparisons and conclusions.

LCS Systems are commonly used in both household alarm and smart home systems. Inaccurate calibrations can lead to these sensors reaching their thresholds inappropriately which could cause false triggers. Underestimating pollutant concentrations could lead to alarms not going off during smoke events, or smart home systems not opening windows at the correct times to increase ventilation. Overestimating could lead to oversensitive alarms and automatic windows activating when the indoor air is relatively clean. As such, it is important to be mindful of both the environment used for calibrating these sensors and the environment in which these sensors will be used in order to reduce calibration model error and reduce false activation or inactivation as a result of inaccurate readings.

## Figures and Tables

**Table 1:** Number of 5 min time resolved measurements for each POD and in each truncation set.

Percent of Data	*n*: POD A	*n*: POD B
100%	28,798	19,246
20%	5759	3849
40%	11,519	7698
60%	17,279	11,547

## Data Availability

The data presented in this study are available on request from the corresponding author due to some reference data integral to the study is owned by the Colorado Department of Public Health and the Environment, and sharing permission will be required before dissemination.

## Supplementary Materials

The following supporting information can be downloaded at https://www.mdpi.com/article/10.3390/s26154988/s1: Figure S1: This figure depicts a logical diagram following the data analysis process used from collecting to figure generation. The flow chart on the left is a simplified process while the flow chart on the right includes more detail to support the methods section of the article. Figure S2: This plot depicts the temperature readings of POD A vs. the PM_2.5_ concentrations of the reference monitor to demonstrate how the two measurements are not independent. As such, when extrapolating in one dimension (temperature) one may be extrapolating in another (PM_2.5_ concentration). This case demonstrates that the highest PM_2.5_ concentrations fall in the lower 40% of temperature measurements, even though measurements from the top 20% of PM_2.5_ concentrations do fall in the highest temperature ranges. When a model trained on the highest temperatures is extrapolated into lower temperature ranges, statistical error in the model performance may be related to differing PM_2.5_ concentrations rather than the impact of temperature on the sensor or model in that temperature range. Table S1: This table is a collection of the performance of all models developed on truncated CO-sorted datasets from both PODs. Each row indicates the respective model as defined by its truncation amount, the pollutant the model is for, the location of the truncation, and which POD the training dataset is taken from. Table S2: This table is a collection of the performance of all models developed on truncated PM_2.5_-sorted datasets from both PODs. Each row indicates the respective model as defined by its truncation amount, the pollutant the model is for, the method of regression, the location of the truncation, and which POD the training dataset is taken from. Table S3: This table is a collection of the performance of all CO models developed on truncated temperature-sorted datasets from both PODs. Each row indicates the respective model as defined by its truncation amount, the pollutant for which the model is trained, the location of the truncation, and which POD the training dataset is taken from. Table S4: This table is a collection of the performance of all PM_2.5_ models developed on truncated temperature-sorted datasets from both PODs. Each row indicates the respective model as defined by its truncation amount, the pollutant for which the model is trained, the location of the truncation, and which POD the training dataset is taken from. Table S5: This table is a collection of the performance of all CO models developed on truncated relative humidity-sorted datasets from both PODs. Each row indicates the respective model as defined by its truncation amount, the pollutant for which the model is trained, the location of the truncation, and which POD the training dataset is taken from. Table S6: This table is a collection of the performance of all PM_2.5_ models developed on truncated relative humidity-sorted datasets from both PODs. Each row indicates the respective model as defined by its truncation amount, the pollutant for which the model is trained, the location of the truncation, and which POD the training dataset is taken from.
