# Supplementary material for: Limitations of Environmental Extrapolation in Low-Cost Carbon Monoxide and Fine Particulate Matter Sensors
Source: Sensors (Basel). 2026 Aug 6;26(15):4988. doi: 10.3390/s26154988 (PMC13469002; doi:10.3390/s26154988)
Supplement: Supplementary file 1 [file sensors-26-04988-s001.zip › Supplemental Figure S1.pdf]

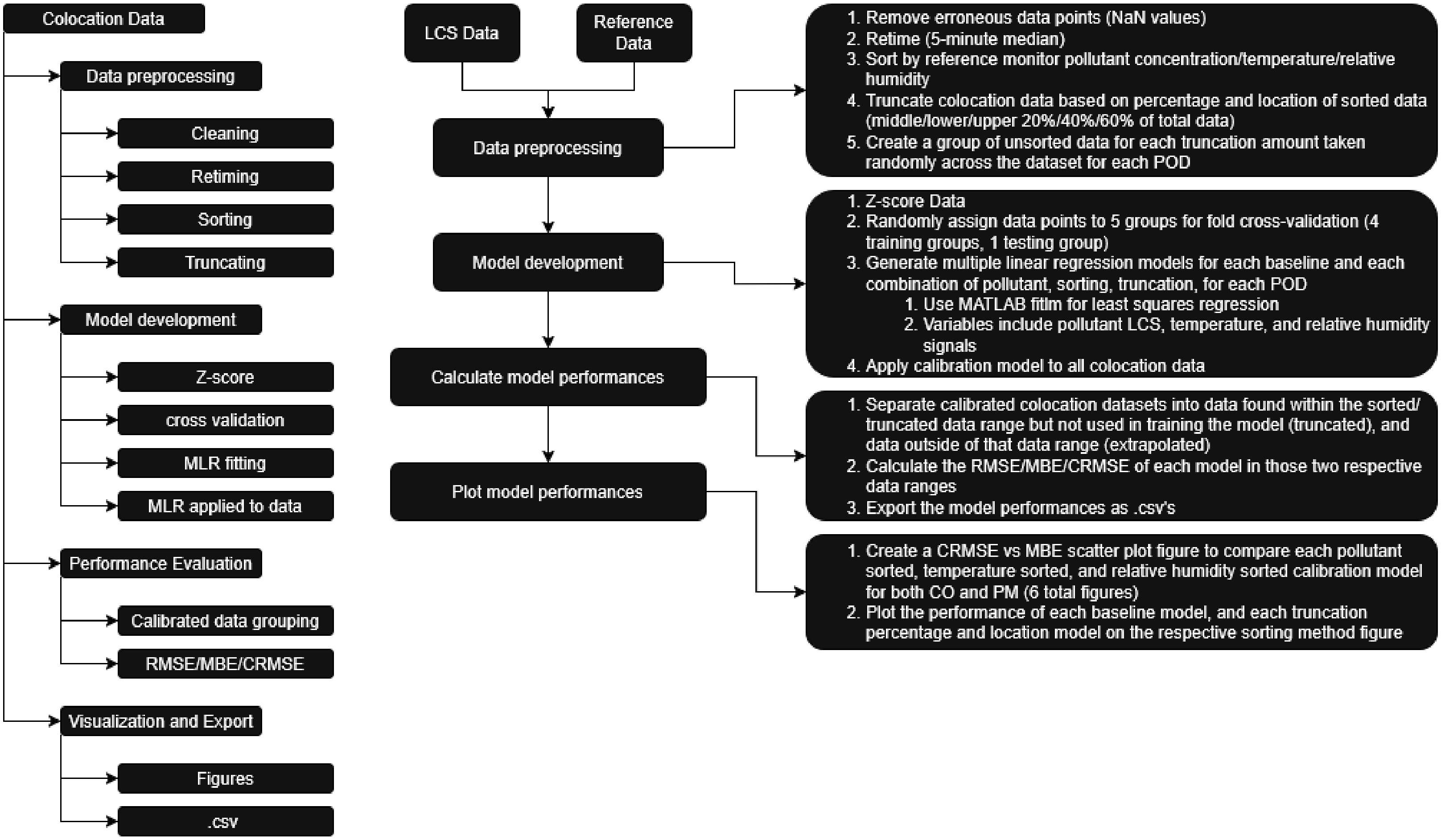

Supplemental Figure S1: This figure depicts a logical diagram following the data analysis process used from collecting to figure generation. The flow chart on the left is a simplified process while the flow chart on the right includes more detail to support the methods section of the article.
