# Supplementary material for: Limitations of Environmental Extrapolation in Low-Cost Carbon Monoxide and Fine Particulate Matter Sensors
Source: Sensors (Basel). 2026 Aug 6;26(15):4988. doi: 10.3390/s26154988 (PMC13469002; doi:10.3390/s26154988)
Supplement: Supplementary file 1 [file sensors-26-04988-s001.zip › Supplemental Figure S2.pdf]

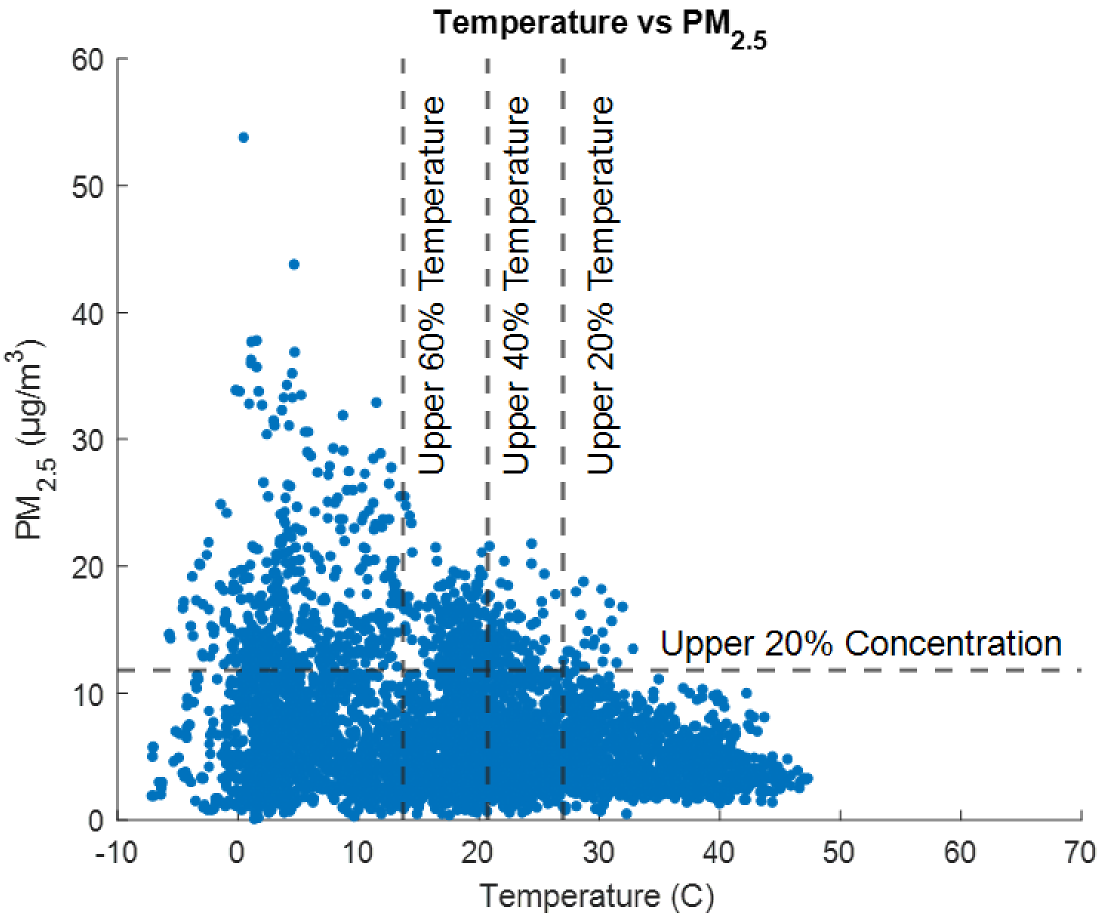

Supplemental Figure S2. This plot depicts the temperature readings of POD A vs the $PM_{2.5}$ concentrations of the reference monitor to demonstrate how the two measurements are not independent. As such, when extrapolating in one dimension (temperature) one may be extrapolating in another ($PM_{2.5}$ concentration). This case demonstrates that the highest $PM_{2.5}$ concentrations fall in the lower 40% of temperature measurements, even though measurements from the top 20% of $PM_{2.5}$ concentrations do fall in the highest temperature ranges. When a model trained on the highest temperatures is extrapolated into lower temperature ranges, statistical error in the model performance may be related to differing $PM_{2.5}$ concentrations rather than the impact of temperature on the sensor or model in that temperature range.
