# Supplementary material for: Limitations of Environmental Extrapolation in Low-Cost Carbon Monoxide and Fine Particulate Matter Sensors
Source: Sensors (Basel). 2026 Aug 6;26(15):4988. doi: 10.3390/s26154988 (PMC13469002; doi:10.3390/s26154988)
Supplement: Supplementary file 1 [file sensors-26-04988-s001.zip › Supplemental Table S1.pdf]

Supplemental Table S1: This table is a collection of the performance of all models developed on truncated CO-sorted datasets from both PODs. Each row indicates the respective model as defined by its truncation amount, the pollutant the model is for, the location of the truncation, and which POD the training dataset is taken from.

Truncation	Pollutant	Group	Pod	Truncated CRMSE	Truncated MBE	Extrapolated CRMSE	Extrapolated MBE
20	CO	Baseline	A	0.117823	0.002842	0.143736	0.018784
20	CO	Baseline	B	0.130396	-0.00119	0.125616	0.004881
20	CO	Lower	A	0.066661	0.00039	0.266387	0.28774
20	CO	Lower	B	0.071154	0.001749	0.287594	0.334247
20	CO	Middle	A	0.078263	0.002066	0.254477	0.072105
20	CO	Middle	B	0.103468	0.001422	0.239588	0.109408
20	CO	Upper	A	0.132665	-0.00298	0.119592	-0.37719
20	CO	Upper	B	0.131645	-0.00283	0.148401	-0.47178
40	CO	Baseline	A	0.118218	-0.00176	0.14424	0.020299
40	CO	Baseline	B	0.125724	0.001112	0.127001	0.004234
40	CO	Lower	A	0.0749	0.000938	0.258603	0.297292
40	CO	Lower	B	0.084651	0.002473	0.26633	0.316949
40	CO	Middle	A	0.077017	-0.00087	0.27436	0.090407
40	CO	Middle	B	0.108774	-0.00017	0.239846	0.108232
40	CO	Upper	A	0.12393	0.00425	0.106532	-0.23747
40	CO	Upper	B	0.134389	-0.00089	0.127865	-0.27475

60	CO	Baseline	A	0.11825	-0.00028	0.142528	0.014971
60	CO	Baseline	B	0.128424	-0.00209	0.123282	0.005407
60	CO	Lower	A	0.076458	6.51E-05	0.263469	0.315484
60	CO	Lower	B	0.092433	-0.00055	0.257531	0.326704
60	CO	Middle	A	0.08524	-0.00029	0.294237	0.113132
60	CO	Middle	B	0.116888	-0.00191	0.245406	0.112543
60	CO	Upper	A	0.119265	-0.00036	0.10471	-0.16163
60	CO	Upper	B	0.130727	-0.00159	0.117168	-0.16177
