# Supplementary material for: Limitations of Environmental Extrapolation in Low-Cost Carbon Monoxide and Fine Particulate Matter Sensors
Source: Sensors (Basel). 2026 Aug 6;26(15):4988. doi: 10.3390/s26154988 (PMC13469002; doi:10.3390/s26154988)
Supplement: Supplementary file 1 [file sensors-26-04988-s001.zip › Supplemental Table S2.pdf]

Supplemental Table S2: This table is a collection of the performance of all models developed on truncated $PM_{2.5}$-sorted datasets from both PODs. Each row indicates the respective model as defined by its truncation amount, the pollutant the model is for, the method of regression, the location of the truncation, and which POD the training dataset is taken from.

Truncation	Pollutant	Model Type	Group	Pod	Truncated CRMSE	Truncated MBE	Extrapolated CRMSE	Extrapolated MBE
20	PM	MLR	Baseline	A	2.177972	-0.03008	5.490179	0.442321
20	PM	MLR	Baseline	B	2.644838	0.090919	3.001662	0.125069
20	PM	MLR	Lower	A	0.921303	-0.00747	5.447297	5.951788
20	PM	MLR	Lower	B	2.18381	-0.03557	5.796968	7.538341
20	PM	MLR	Middle	A	1.212703	-0.0371	5.958364	1.975271
20	PM	MLR	Middle	B	2.298503	0.0506	4.846624	1.790347
20	PM	MLR	Upper	A	3.125631	-0.03439	3.262231	-9.56237
20	PM	MLR	Upper	B	2.909114	0.064076	3.351942	-10.6301
40	PM	MLR	Baseline	A	2.17658	0.043261	5.518043	0.544793
40	PM	MLR	Baseline	B	2.750395	0.013783	2.926882	-0.03925
40	PM	MLR	Lower	A	1.09092	0.031667	5.50463	6.644885
40	PM	MLR	Lower	B	1.9114	0.022328	5.147616	8.044739
40	PM	MLR	Middle	A	1.452469	-0.00129	6.615573	2.387956
40	PM	MLR	Middle	B	2.351014	0.025269	5.15039	1.994053
40	PM	MLR	Upper	A	2.692163	0.023962	2.517602	-7.23612
40	PM	MLR	Upper	B	2.887985	-0.14007	2.885047	-6.85917

60	PM	MLR	Baseline	A	2.12846	-0.07295	5.415048	0.563693
60	PM	MLR	Baseline	B	2.681483	0.009651	2.870615	-0.01112
60	PM	MLR	Lower	A	1.429413	0.014823	5.612428	7.85819
60	PM	MLR	Lower	B	2.166262	-0.01104	4.254907	7.557626
60	PM	MLR	Middle	A	1.668909	0.015436	7.59944	2.906566
60	PM	MLR	Middle	B	2.637667	-0.00383	4.991263	1.934399
60	PM	MLR	Upper	A	2.501532	0.092465	2.357868	-6.02447
60	PM	MLR	Upper	B	2.796066	0.105869	2.926827	-4.43108
60	PM	GB	Lower	A	0.870629	-0.01665	4.753017	5.782373
60	PM	GB	Middle	A	1.190111	0.027012	4.75239	2.329651
60	PM	GB	Upper	A	2.917863	0.00359	2.284232	-1.45609
