# Supplementary material for: Limitations of Environmental Extrapolation in Low-Cost Carbon Monoxide and Fine Particulate Matter Sensors
Source: Sensors (Basel). 2026 Aug 6;26(15):4988. doi: 10.3390/s26154988 (PMC13469002; doi:10.3390/s26154988)
Supplement: Supplementary file 1 [file sensors-26-04988-s001.zip › Supplemental Table S3.pdf]

Supplemental Table S3: This table is a collection of the performance of all CO models developed on truncated temperature-sorted datasets from both PODs. Each row indicates the respective model as defined by its truncation amount, the pollutant for which the model is trained, the location of the truncation, and which POD the training dataset is taken from.

Truncation	Pollutant	Group	Pod	Truncated CRMSE	Truncated MBE	Extrapolated CRMSE	Extrapolated MBE
20	CO	Baseline	A	0.117823	0.002842	0.143736	0.018784
20	CO	Baseline	B	0.130396	-0.00119	0.125616	0.004881
20	CO	Lower Temp	A	0.085193	0.000802	0.119371	-0.09711
20	CO	Lower Temp	B	0.062192	0.002467	0.153696	0.064387
20	CO	Middle Temp	A	0.081284	-0.00147	0.189402	0.068872
20	CO	Middle Temp	B	0.087292	-0.00151	0.174452	-0.05442
20	CO	Upper Temp	A	0.090117	-0.00185	0.217529	0.132891
20	CO	Upper Temp	B	0.108884	-0.00039	0.196516	0.16028
40	CO	Baseline	A	0.117634	-9.93E-05	0.14424	0.020299
40	CO	Baseline	B	0.124602	0.001838	0.127001	0.004234
40	CO	Lower Temp	A	0.092314	-0.00207	0.117991	-0.10264
40	CO	Lower Temp	B	0.089674	-0.00188	0.154722	-0.01118

40	CO	Middle Temp	A	0.091251	0.002996	0.170397	0.059353
40	CO	Middle Temp	B	0.092808	-0.00204	0.170293	-0.06059
40	CO	Upper Temp	A	0.088644	-0.00324	0.226971	0.142948
40	CO	Upper Temp	B	0.124906	-0.00091	0.129421	0.065152
60	CO	Baseline	A	0.116153	0.000455	0.142528	0.014971
60	CO	Baseline	B	0.12765	0.00265	0.123282	0.005407
60	CO	Lower Temp	A	0.096484	-0.00084	0.11584	-0.04463
60	CO	Lower Temp	B	0.091356	-0.00097	0.173891	-0.03749
60	CO	Middle Temp	A	0.097878	0.000705	0.158713	0.041598
60	CO	Middle Temp	B	0.110443	0.001453	0.153147	-0.04657
60	CO	Upper Temp	A	0.092943	0.000815	0.24209	0.166691
60	CO	Upper Temp	B	0.123964	0.006619	0.117178	0.059167
