# Supplementary material for: Limitations of Environmental Extrapolation in Low-Cost Carbon Monoxide and Fine Particulate Matter Sensors
Source: Sensors (Basel). 2026 Aug 6;26(15):4988. doi: 10.3390/s26154988 (PMC13469002; doi:10.3390/s26154988)
Supplement: Supplementary file 1 [file sensors-26-04988-s001.zip › Supplemental Table S4.pdf]

Supplemental Table S4: This table is a collection of the performance of all $PM_{2.5}$ models developed on truncated temperature-sorted datasets from both PODs. Each row indicates the respective model as defined by its truncation amount, the pollutant for which the model is trained, the location of the truncation, and which POD the training dataset is taken from.

Truncation	Pollutant	Group	Pod	Truncated CRMSE	Truncated MBE	Extrapolated CRMSE	Extrapolated MBE
20	PM	Baseline	A	2.210122	0.029778	5.490179	0.442321
20	PM	Baseline	B	2.636269	0.013578	3.001662	0.125069
20	PM	Lower Temp	A	2.37189	-0.03582	5.273609	-2.22717
20	PM	Lower Temp	B	1.74585	0.027099	2.982766	-0.20067
20	PM	Middle Temp	A	1.480653	-0.00459	5.637011	0.467014
20	PM	Middle Temp	B	2.23965	0.044904	3.020646	-0.5403
20	PM	Upper Temp	A	1.533834	-0.00415	5.6028	2.517442
20	PM	Upper Temp	B	2.613152	0.059099	3.431291	2.896353
40	PM	Baseline	A	2.249841	0.022523	5.518043	0.544793
40	PM	Baseline	B	2.983489	0.024085	2.926882	-0.03925
40	PM	Lower Temp	A	2.420529	-0.07234	4.450859	-2.77878
40	PM	Lower Temp	B	2.54626	-0.08772	2.797804	-0.44048

40	PM	Middle Temp	A	1.659229	0.113554	5.650663	0.618421
40	PM	Middle Temp	B	2.927507	-0.13527	2.936735	-1.09005
40	PM	Upper Temp	A	1.590893	-0.00473	6.111608	2.476909
40	PM	Upper Temp	B	2.730836	0.004551	3.024482	0.571077
60	PM	Baseline	A	2.300721	0.074839	5.415048	0.563693
60	PM	Baseline	B	2.978952	0.066706	2.870615	-0.01112
60	PM	Lower Temp	A	2.374036	0.014496	3.621986	-2.95433
60	PM	Lower Temp	B	2.314057	-0.00764	2.974283	-0.89709
60	PM	Middle Temp	A	1.857616	0.020542	5.838435	0.562878
60	PM	Middle Temp	B	2.945289	-0.04609	2.857424	-1.45955
60	PM	Upper Temp	A	1.596672	-0.03135	6.661107	2.323956
60	PM	Upper Temp	B	2.667056	-0.01524	2.942141	-0.30428
