# Supplementary material for: Limitations of Environmental Extrapolation in Low-Cost Carbon Monoxide and Fine Particulate Matter Sensors
Source: Sensors (Basel). 2026 Aug 6;26(15):4988. doi: 10.3390/s26154988 (PMC13469002; doi:10.3390/s26154988)
Supplement: Supplementary file 1 [file sensors-26-04988-s001.zip › Supplemental Table S5.pdf]

Supplemental Table S5: This table is a collection of the performance of all CO models developed on truncated relative humidity-sorted datasets from both PODs. Each row indicates the respective model as defined by its truncation amount, the pollutant for which the model is trained, the location of the truncation, and which POD the training dataset is taken from.

Truncation	Pollutant	Group	Pod	Truncated CRMSE	Truncated MBE	Extrapolated CRMSE	Extrapolated MBE
20	CO	Baseline	A	0.117823	0.002842	0.143736	0.018784
20	CO	Baseline	B	0.130396	-0.00119	0.125616	0.004881
20	CO	Lower RH	A	0.090281	0.001127	0.216531	0.135753
20	CO	Lower RH	B	0.108506	-0.00243	0.213685	0.178738
20	CO	Middle RH	A	0.095988	-0.00419	0.148332	0.004064
20	CO	Middle RH	B	0.11964	-0.00116	0.141789	-0.0878
20	CO	Upper RH	A	0.080639	-0.00023	0.123469	-0.01323
20	CO	Upper RH	B	0.070848	-0.0028	0.149021	0.035318
40	CO	Baseline	A	0.117634	-9.93E-05	0.14424	0.020299
40	CO	Baseline	B	0.124602	0.001838	0.127001	0.004234
40	CO	Lower RH	A	0.104003	0.000988	0.211896	0.12233
40	CO	Lower RH	B	0.131808	-0.00129	0.16719	0.097746

40	CO	Middle RH	A	0.102363	-0.00256	0.14986	0.008135
40	CO	Middle RH	B	0.126412	-0.00577	0.144538	-0.06955
40	CO	Upper RH	A	0.092274	-0.00057	0.11859	-0.04468
40	CO	Upper RH	B	0.084243	-0.00066	0.176398	-0.04146
60	CO	Baseline	A	0.116153	0.000455	0.142528	0.014971
60	CO	Baseline	B	0.12765	0.00265	0.123282	0.005407
60	CO	Lower RH	A	0.107469	0.00202	0.201684	0.108784
60	CO	Lower RH	B	0.132619	0.003061	0.136867	0.0216
60	CO	Middle RH	A	0.112408	0.001859	0.138144	0.00094
60	CO	Middle RH	B	0.121808	0.003773	0.148922	-0.0461
60	CO	Upper RH	A	0.094786	0.001225	0.119235	-0.01171
60	CO	Upper RH	B	0.097917	0.000943	0.194275	-0.05576
