# Supplementary material for: Limitations of Environmental Extrapolation in Low-Cost Carbon Monoxide and Fine Particulate Matter Sensors
Source: Sensors (Basel). 2026 Aug 6;26(15):4988. doi: 10.3390/s26154988 (PMC13469002; doi:10.3390/s26154988)
Supplement: Supplementary file 1 [file sensors-26-04988-s001.zip › Supplemental Table S6.pdf]

Supplemental Table S6: This table is a collection of the performance of all $PM_{2.5}$ models developed on truncated RH-sorted datasets from both PODs. Each row indicates the respective model as defined by its truncation amount, the pollutant for which the model is trained, the location of the truncation, and which POD the training dataset is taken from.

Truncation	Pollutant	Group	Pod	Truncated CRMSE	Truncated MBE	Extrapolated CRMSE	Extrapolated MBE
20	PM	Baseline	A	2.210122	0.029778	5.490179	0.442321
20	PM	Baseline	B	2.636269	0.013578	3.001662	0.125069
20	PM	Lower RH	A	1.36665	-0.04406	5.575132	3.565861
20	PM	Lower RH	B	2.400627	-0.0741	3.993414	3.799533
20	PM	Middle RH	A	1.825038	-0.01468	5.187489	-0.32202
20	PM	Middle RH	B	2.298501	-0.16477	2.861215	-0.00431
20	PM	Upper RH	A	2.505983	-0.04406	4.993284	-2.41106
20	PM	Upper RH	B	1.961225	0.017187	2.998801	-1.88234
40	PM	Baseline	A	2.249841	0.022523	5.518043	0.544793
40	PM	Baseline	B	2.983489	0.024085	2.926882	-0.03925
40	PM	Lower RH	A	1.634963	0.044566	5.982313	3.156062
40	PM	Lower RH	B	2.867625	0.246854	3.009463	1.758772

40	PM	Middle RH	A	1.865745	-0.0156	5.378304	-0.09062
40	PM	Middle RH	B	2.828623	-0.02574	2.820459	-0.56591
40	PM	Upper RH	A	2.347487	-0.02229	4.46928	-3.02595
40	PM	Upper RH	B	2.107155	-0.03812	3.176172	-1.6649
60	PM	Baseline	A	2.300721	0.074839	5.415048	0.563693
60	PM	Baseline	B	2.978952	0.066706	2.870615	-0.01112
60	PM	Lower RH	A	1.81636	0.02001	6.311756	2.451916
60	PM	Lower RH	B	2.80961	-0.03434	2.53038	0.898873
60	PM	Middle RH	A	1.91301	0.02397	5.616478	-0.03009
60	PM	Middle RH	B	2.826399	-0.02264	2.784753	-1.0328
60	PM	Upper RH	A	2.260267	-0.02154	3.326442	-3.80035
60	PM	Upper RH	B	2.649177	-0.04507	3.138569	-1.51961
